# Anthocyanins Double the Shelf Life of Tomatoes by Delaying Overripening and Reducing Susceptibility to Gray Mold

**DOI:** 10.1016/j.cub.2013.04.072

**Published:** 2013-06-17

**Authors:** Yang Zhang, Eugenio Butelli, Rosalba De Stefano, Henk-jan Schoonbeek, Andreas Magusin, Chiara Pagliarani, Nikolaus Wellner, Lionel Hill, Diego Orzaez, Antonio Granell, Jonathan D.G. Jones, Cathie Martin

**Affiliations:** 1 John Innes Centre, Norwich Research Park, Norwich, NR4 7UH, UK; 2 Department of Soil, Plant, Environmental, and Animal Sciences, University of Naples Federico II, 80055 Portici, Italy; 3 Department of Agricultural, Forestry, and Food Sciences, University of Turin, via Leonardo da Vinci 44, 10095 Grugliasco TO, Italy; 4 Institute of Food Research, Norwich Research Park, Colney, Norwich, NR4 7UA, UK; 5 Instituto de Biología Molecular y Celular de Plantas, Consejo Superior de Investigaciones Científicas-Universidad Politécnica de Valencia, 46022 Valencia, Spain; 6 The Sainsbury Laboratory, Norwich Research Park, Colney, Norwich, NR4 7UH, UK

## Abstract

Shelf life is an important quality trait for many fruit, including tomatoes. We report that enrichment of anthocyanin, a natural pigment, in tomatoes can significantly extend shelf life. Processes late in ripening are suppressed by anthocyanin accumulation, and susceptibility to *Botrytis cinerea*, one of the most important postharvest pathogens, is reduced in purple tomato fruit. We show that reduced susceptibility to *B. cinerea* is dependent specifically on the accumulation of anthocyanins, which alter the spreading of the ROS burst during infection. The increased antioxidant capacity of purple fruit likely slows the processes of overripening. Enhancing the levels of natural antioxidants in tomato provides a novel strategy for extending shelf life by genetic engineering or conventional breeding.

## Highlights

• Anthocyanin accumulation doubles the postharvest storage time of tomato fruit
• Anthocyanin enrichment reduces susceptibility of tomato fruit to *Botrytis cinerea*
• Longer shelf life is associated with high hydrophilic antioxidant capacity of fruit
• Anthocyanins perturb the dynamics of the ROS burst during infection by *B. cinerea*

## Graphical Abstract

**Figure:**
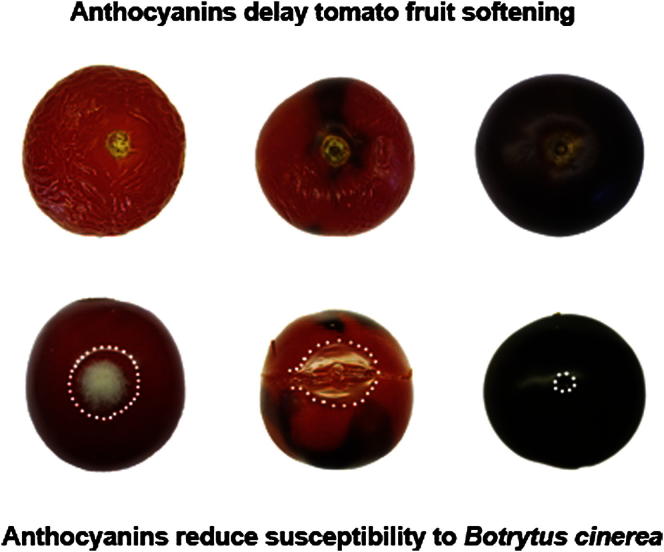

## Results and Discussion

Important challenges for the cultivation of tomatoes include postharvest losses and reduced quality due to fruit senescence and pathogen infection. Many tomatoes grown for fresh consumption are picked when still firm and green, stored at low temperature, and exposed to exogenous ethylene to induce color and ripeness before reaching the supermarket shelf. Although effective in limiting postharvest losses, these procedures negatively affect tomato flavor, aroma, and texture [1]. The common use of mutants affected in ripening has similar negative impacts on flavor. Over the last two decades, genetic engineering has been used to extend tomato shelf life by reducing the activity of cell-wall-degrading enzymes [2–5] and enhancing the levels of specific metabolites [6, 7].

Anthocyanins are water-soluble pigments responsible for the red, purple, and blue colors of many flowers and fruit [8]. They are produced by plants to attract pollinators and seed dispersers [9]. Anthocyanin production is also commonly induced under stress conditions [10] and infection by pathogens [11]. Besides physiological roles in plants, dietary anthocyanins are associated with protection against certain cancers [12], cardiovascular diseases [13], and other chronic human disorders [13].

**Figure 1:**
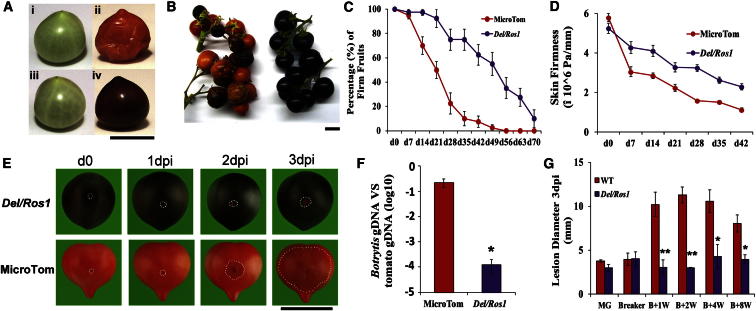
Accumulation of Anthocyanins in Tomato Fruit Delays Late Ripening and Decreases Pathogen Susceptibility (A) Wild-type, red (i and ii) and transgenic, purple (iii and iv) tomato fruits were tagged during the initial stages of development and harvested and photographed at the end of the green stage (i and iii). The same fruit, stored at room temperature, was rephotographed after 2 months (ii and iv). The scale bar represents 2 cm. (B) Severe symptoms of opportunistic infection normally associated with overripe red, wild-type tomato fruit on the vine (left) were not observed in purple, *Del/Ros1* tomato fruit of the same age grown under identical greenhouse conditions (right). The scale bar represents 2 cm. (C) Purple fruit showed slower softening as determined by visual inspection compared to wild-type, red fruit. Percentages of fruit showing overripening symptoms were assessed every week during storage tests. Error bars show the SEM (n = 4). Fruits were harvested at 14 days postbreaker (d0 = 14 dpb). (D) Texture strength changes in MicroTom and *Del/Ros1* fruits during storage tests. Average values were calculated for at least eight individual fruits, and error bars indicate the SEM. (E) Symptoms of wounded red and purple fruits after inoculation with *B. cinerea* B05.10. White dots represent the lesion margins. (F) Quantitative PCR revealed more *Botrytis* growing on the WT tomatoes than on purple fruit, 3 dpi. *Botrytis* growth was calculated by comparison of the ratio of *Botrytis* DNA to tomato DNA. Error bars show the SEM (n = 3). ^∗^p < 0.05 compared to control red tomato. (G) The ripening-related increase in susceptibility to *Botrytis* did not occur in purple fruit. Lesion diameter was measured 3 dpi. Error bars show the SEM (n ≥ 3). The scale bar represents 2 cm. ^∗^p < 0.05 and ^∗∗^p < 0.01 for values for purple tomatoes compared to red tomatoes at the same stage. See also Figures S1 and S2.

We have shown that ectopic expression of two genes encoding transcription factors, Delila (Del) and Rosea1 (Ros1), from snapdragons, under the control of the fruit-specific E8 promoter, results in increased expression of all the genes committed to anthocyanin biosynthesis to create intensely purple tomato fruit [14]. While growing the purple tomatoes, we observed that they had improved shelf life compared to wild-type, red fruit. The shelf life of food is defined as the period during which a stored product remains suitable for consumption and is normally determined by the degree of softening, shriveling, and rotting of fruit. Consequently, both fruit softening late during ripening and pathogen infection influence the shelf life of tomatoes. Purple fruit from *Del/Ros1* tomato plants have normal size, shape, and number of seeds. However, purple fruit exhibit delayed ripening after breaker compared to red fruit. This is evident from the appearance of the purple fruit both on the vine and during postharvest storage and from a reduced level of fungal infection under either condition (Figures 1A and 1B).

Both wild-type (WT) and purple tomatoes were harvested when ripe and stored under sterile conditions. For purple fruit, 49 days of storage at 18°C were required to observe 50% of the fruit softened, equivalent to the level of softening observed in red fruit at 21 days. Complete collapse was observed in purple fruit after 10 weeks storage, compared to 5 weeks for red fruit. With a texture analyzer, the firmness of red fruit was measured as 50% lower than that at breaker, after 2 weeks at 18°C, whereas the same reduction in firmness was reached after 5 weeks storage of purple fruit. These results indicated that expression of *Del* and *Ros1* can more than double the shelf life of tomato fruit (Figure 1C). These differences were accompanied by greater ability to resist tensile forces in purple tomatoes compared to red fruit of equivalent age (Figure 1D).

Production of ethylene, required for full ripening in climacteric fruit such as tomato, increased just after breaker and was 2-fold greater in purple fruit than in red fruit (Figure S1A available online). Measurements of cuticle thickness revealed no differences between WT and purple tomato (Figures S1B–S1D). In addition, Fourier transform infrared (FT-IR) spectroscopy indicated that there were no significant cell wall compositional differences between purple tomato peel and red tomato peel 1 week after breaker (Figure S2E). These observations implied that the extended shelf life of purple fruit was due to neither impaired ethylene production nor altered cuticle/peel composition.

The susceptibility of purple fruit to pathogens was investigated by infection of intact or wounded tomatoes with *B. cinerea*, the causal agent of gray mold disease, one of the most important postharvest pathogens of tomatoes [15]. When intact fruit were sprayed with a *B. cinerea* spore suspension without wounding, the proportion of purple fruit showing severe symptoms of infection was substantially lower than for red fruit (Figures S2A and S2B). When wounded fruit were inoculated with the *B. cinerea* spore suspension, the size of the lesions did not increase 1 day postinoculation (dpi) in either fruit type, indicating that the fungus needs about 24 hr to establish after inoculation. From 2 dpi, however, there was greater spread of infection in red fruit than in purple fruit. At 3 dpi, the average size of the lesions in purple tomatoes was significantly smaller than in red fruit, indicating reduced susceptibility to *B. cinerea* infection (Figure 1E). Quantitative PCR with DNA extracted from infected tomatoes confirmed that there was significantly more *Botrytis* growing on red fruit than on purple fruit at 3 dpi (Figure 1F). Reduced pathogen susceptibility was also observed in purple fruit introgressed into the MoneyMaker genetic background (Figure S2C), indicating that the lower susceptibility of purple tomatoes to *B. cinerea* is not dependent on a specific genetic background.

The susceptibility of tomato fruit to necrotrophic pathogens increases during ripening [16, 17]. A correlation between fruit age late in ripening and increased susceptibility was observed in red fruit. However, in purple fruit, susceptibility to *B. cinerea* did not increase from the breaker stage when anthocyanin production was induced (Figure 1G). This observation suggested a specific role for anthocyanins in limiting the spread of fungal infection, as supported by the intermediate susceptibility displayed by two different *Del/Ros1* lines (C and Y) that produce lower levels of anthocyanins than line N (used for the initial tests) [14] (Figures S2D–S2F).

**Figure 2:**
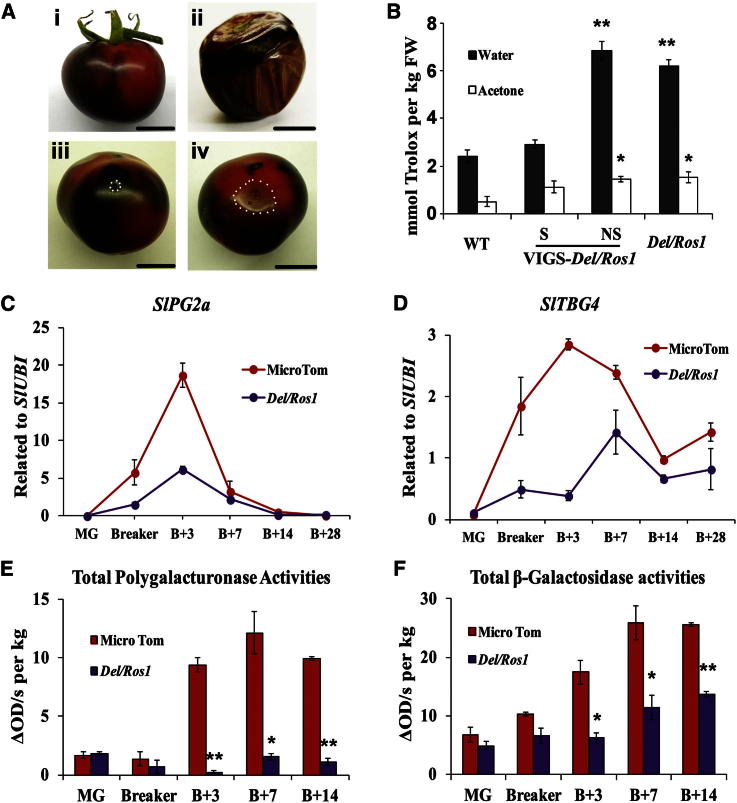
Delayed Ripening and Reduced Pathogen Susceptibility Are Associated with the Accumulation of Anthocyanins, and Expression of Ripening-Related Genes Is Suppressed in Purple Tomatoes (A) VIGS-*Del/Ros1*tomato fruits showed reduced accumulation of anthocyanins in silenced areas (i, pictures taken 14 days after breaker). The red sectors showed quicker softening than purple sectors (ii, pictures taken 42 days after breaker). Purple sectors showed reduced susceptibility to *B. cinerea* 3 dpi (iii). Red sectors of VIGS-silenced tomatoes were more susceptible to *B. cinerea* 3 dpi (iv). All scale bars represent 2 cm. (B) VIGS-*Del/Ros1* silenced sectors had reduced hydrophilic antioxidant capacity compared to purple sectors. Error bars show the SEM (n = 3). S, silenced sectors; NS, nonsilenced sectors. ^∗^p < 0.05 and ^∗∗^p < 0.01 compared to the WT. (C and D) Quantitative RT-PCR analysis of genes encoding cell-wall-modifying enzymes in WT and *Del/Ros1* fruits during ripening. Polygalacturonase 2a (*SlPG2a*) (C) and β-galactosidase 4 (*SlTBG4*) (D) are shown. Error bars show the SEM (n = 3). (E and F) Total polygalacturonase (E) and β-galactosidase (F) activities in red and purple fruit at different stages during ripening. Error bars show the SEM (n = 3). ^∗^p < 0.05 and ^∗∗^p < 0.01 compared to the WT at the same stage. See also Figure S3, Table S1, and Data Set S1.

To ensure that the effects on delayed ripening and pathogen susceptibility were compared at exactly the same developmental stage, we used virus-induced gene silencing (VIGS) to silence the expression of *Del* and *Ros1* in purple fruit in the MoneyMaker background (in which large fruit size allows dissection of tissue sectors relatively easily). Agro-infiltrated *Del/Ros1* fruit showed a phenotype of purple and red sectors, the latter defining those parts of the fruit where *Del* and *Ros1* had been silenced [18] and hydrophilic antioxidant capacity was reduced (Figures 2A and 2B). In older fruit, the red sectors were clearly softer and the tissues were more collapsed than in purple sectors, demonstrating the shorter storage life of red sectors compared to purple sectors (Figure 2A). Red sectors also showed greater susceptibility to *B. cinerea* than purple sectors (Figure 2A).

Gene expression profiles of red and purple sectors of VIGS-*Del/Ros1* fruit were compared. Samples were harvested at 8, 30, and 45 days after breaker. A 3-fold difference in expression levels (purple versus red) was set as the threshold for significant changes detected using the TOM2 microarray. Two hundred and forty one genes showed significant differences in expression between purple and red sectors over at least two stages (Figure S3A). Functional annotation revealed that many of these genes are involved in primary and secondary metabolism, cell wall modification, oxidative stress, and pathogen resistance (Figures S3B and S3C and Data Set S1). Reduced expression of many genes known to be involved in overripening was observed in purple sectors (Figures S3B and S3C), indicating that the suppression of expression of these genes in purple tomatoes contributes to the extended shelf life of the fruit.

The suppression of genes involved in overripening in purple fruit was confirmed by quantitative RT-PCR. Genes encoding polygalacturonase (*SlPG2a*) [4] and β-galactosidase (*SlTBG4*) [5], involved in cell wall softening, showed substantially lower expression in purple fruit during ripening (Figures 2C and 2D). The lower levels of gene expression resulted in lower total activities of polygalacturonase and β-galactosidase in purple tomatoes compared to red tomatoes (Figures 2E and 2F). Although the silencing of the individual genes might only have minor effects on softening [3, 5], the combined suppression of a number of different cell wall modification enzymes likely reduces significantly the rate of fruit softening.

**Figure 3:**
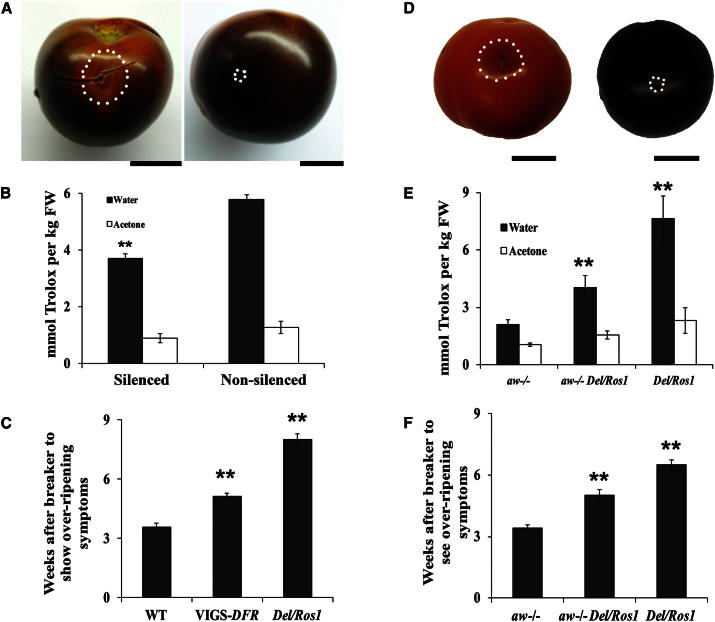
Inhibition of Anthocyanin Biosynthesis in Purple Tomatoes Alters Susceptibility to *Botrytis* and Postharvest Storage (A) VIGS-*SlDFR* silenced sectors had increased susceptibility to *B. cinerea* compared to nonsilenced sectors on the same fruit. Pictures were taken at 3 dpi. White dots indicate lesion sizes. Scale bars represent 2 cm. (B) The hydrophilic antioxidant capacity of VIGS-*SlDFR*-silenced sectors was lower than that of nonsilenced sectors, although still higher than that of WT fruit due to the accumulation of flavonols. Error bars show the SEM, n = 3. ^∗∗^p < 0.01 for differences in TEAC values of hydrophilic extracts of silenced and nonsilenced tissues. (C) Storage tests indicate VIGS-*SlDFR*-silenced fruit can be kept for longer than WT fruit but for less time than nonsilenced purple fruit. Fruits were harvested 2 weeks after breaker, and the times to show overripening symptoms (visual rotting and collapse of fruit) were recorded. Error bars show the SEM, n = 7. ^∗∗^p < 0.01 compared with WT, red fruit. (D) High levels of flavonols accumulate in *aw*^*−/−*^*Del/Ros1* F2 tomato fruit obtained by crossing *Del/Ros1*MicroTom with *aw*^*−/−*^ (*DFR*^*–*^) mutants. The orange, flavonols-enriched tomato (left) was more susceptible to *B. cinerea*. Pictures were taken at 3 dpi. White dots show lesion boundries. Scale bars represent 2 cm. (E) Comparisons of antioxidant capacities of *aw*^*−/−*^*,aw*^*−/−*^*Del/Ros1*and *Del/Ros1*fruit. Error bars show the SEM, n = 3. Solid bars show hydrophilic antioxidant capacity, and open bars show lipophilic antioxidant capacity. ^∗∗^p < 0.01 compared with parental *aw*^*−/−*^ fruit. (F) *aw*^*−/−*^*Del/Ros1* fruit had a longer shelf life than parental *aw*^*−/−*^ fruit but a shorter shelf life than *Del/Ros1* fruit. Times (after breaker) for fruit to show overripening symptoms (visual rotting and collapse of fruit) were recorded. Error bars show the SEM, n = 10. ^∗∗^p < 0.01 compared with parental *aw*^*−/−*^ fruit. See also Figure S4.

To identify specific effects of anthocyanins on extension of shelf life, we silenced dihydroflavonol 4-reductase (*SlDFR*), a key gene in anthocyanin biosynthesis, using VIGS in purple tomatoes. On the same fruit, VIGS-*SlDFR*-silenced, orange sectors showed similar expression levels of *Del* and *Ros1* to nonsilenced, purple sectors, whereas *SlDFR* expression was substantially reduced (Figure S4A). Anthocyanin levels were reduced by 80%, although other flavonoids accumulated in the silenced sectors, giving them an orange color (Figures S4B and S4C). *SlDFR*-silenced sectors were sensitive to *B. cinerea*, whereas purple sectors on the same fruit remained resistant (Figure 3A). Compared to nonsilenced sectors, *SlDFR*-silenced sectors had reduced hydrophilic antioxidant capacity (Figure 3B), although this was higher than the hydrophilic antioxidant capacity of WT red fruit, due to the accumulation of flavonols. Storage tests indicated that VIGS-*SlDFR*-silenced fruit could be kept longer than WT fruit but not as long as purple tomatoes (Figure 3C). We confirmed these observations by crossing *Del/Ros1* plants to the *aw* mutant of tomato in the Ailsa Craig genetic background, which lacks DFR activity and cannot make anthocyanins [19]. In the F2, the plants that contained *Del/Ros1* but lacked DFR activity (*aw*^−/−^) produced orange fruit due to high levels of flavonols. Like the VIGS-*SlDFR*-silenced sectors, the *aw*^−/−^*, Del/Ros1* fruit were no less susceptible to *B. cinerea* than were red tomatoes (Figure 3D). The orange fruit had 2-fold higher hydrophilic antioxidant capacity than the parental *aw*^*−/−*^ line (Figure 3E) and they could be kept longer postharvest, although not as long as purple tomatoes (Figure 3F). Consequently, the delay in overripening and the enhanced pathogen resistance of purple tomatoes are not due to off-targets of the Del and Ros1 transcription factors. Resistance to *B. cinerea* is specifically the result of the accumulation of anthocyanins, whereas the delay in overripening is most likely associated with the increased hydrophilic antioxidant capacity of the fruit.

Levels of oxidative stress increase markedly in the later stages of ripening and may facilitate many of the metabolic changes associated with maturation of tomato fruit [20]. Comparison of a cultivar with shorter shelf life to one with longer shelf life showed reduced scavenging ability and increased levels of reactive oxygen species (ROS) [21]. Accordingly, increase of antioxidant capacity or reduction of levels of ROS with different antioxidants can extend shelf life [6, 22, 23]. Taken together, our data suggest that elevation of the levels of antioxidants in fruit reduces the tissue-damaging activity of oxidative stress and thus is the most likely cause of the delay in overripening observed in purple (*Del/Ros1*) and orange (VIGS-*SlDFR* and *Del/Ros1,aw*^−/−^) tomatoes.

**Figure 4:**
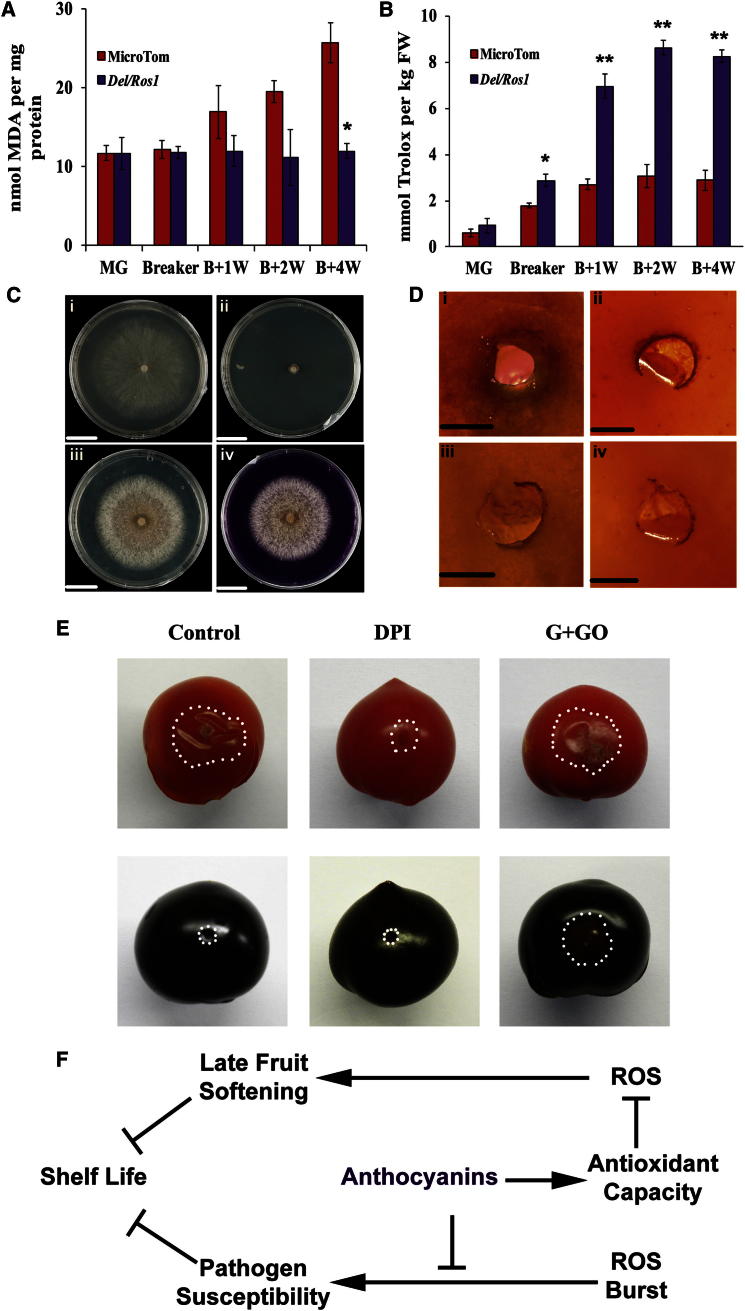
Extended Shelf Life in Purple Tomatoes Is Associated with Their High Antioxidant Capacity (A) Malondialdehyde levels in pericarp of red and purple Microtom fruit during ripening. Error bars show the SEM (n = 3). ^∗^p < 0.05 compared with WT, red fruit at same stage (B) Trolox equivalent total antioxidant capacity (TEAC) of water extracts from red and purple tomatoes during ripening. Error bars show the SEM (n = 3). ^∗^p < 0.05 and ^∗∗^p < 0.01 in comparison to WT, red fruit at the same stages. (C) Addition of juice from either red or purple tomatoes to the growth medium had no effect on growth of *B. cinerea*. PDA medium (i), PDA with 15 mg/liter Triademinol (an inhibitor of fungal growth) (ii), PDA supplemented with 50% red juice (iii), and PDA supplemented with 50% purple juice (iv) are shown. Pictures were taken 3 days after plate inoculation. Scale bars represent 2 cm. (D) 3,3′-diaminobenzidine (DAB) staining of hydrogen peroxide produced 24 hr after inoculation of *B. cinerea*: red (i) and purple (ii) fruits stained with DAB, 24 hr after inoculation, wound only red (iii) and purple (iv) fruit stained 24 hr after wounding. Scale bars represent 1 mm. (E) The levels of ROS in red and purple tomatoes were altered by infiltration of a water control, 10 mM diphenyleneiodonium chloride (DPI, ROS inhibitor), or 50 units/ml glucose oxidase plus 1% glucose (G+GO, ROS inducer). Fruits were wounded and infiltrated 1 hr prior to *B. cinerea* inoculation. Pictures were taken 3 dpi. White dotted lines represent lesion margin. All scale bars represent 2 cm. (F) Model for the mechanism of shelf life extension in purple, high-anthocyanin tomatoes.

Malondialdehyde (MDA) is a byproduct of lipid peroxidation and can be used to measure damage resulting from oxidative stress during tissue senescence [21, 24]. MDA levels in red MicroTom fruit increased late in ripening. In purple tomatoes, however, MDA levels did not increase significantly up to 4 weeks after breaker (Figure 4A). Lower oxidative damage in purple tomato was associated closely with increased total antioxidant capacity during overripening, which resulted principally from the accumulation of anthocyanins (Figure 4B). Higher hydrophilic antioxidant capacity/lower ROS levels were associated with suppression of ripening-related enzyme activities such as polygalacturonase and β-galactosidase, an effect likely to be of importance in extending shelf life, since downregulation of some of the corresponding genes by antisense has been shown to result in fruit that are firmer for longer than controls [3, 5] and their combined suppression may extend shelf life yet further. One explanation for the induced expression of these genes, late in ripening, is that it is the result of increased ROS signaling. Our data suggest that ROS signaling is an important determinant of the rate of ripening, late in fruit development. High hydrophilic antioxidant capacity can suppress both ROS activity and signaling and consequently may delay the processes of overripening, both directly and indirectly.

Reduced susceptibility to *B. cinerea* is associated specifically with anthocyanin accumulation. Anthocyanin levels have been associated with reduced susceptibility to *Botrytis* in grapes [25] and may reduce postharvest spoilage of fruits in general by *Botrytis*. When we grew *B. cinerea* on agar plates supplemented with red and purple fruit juice, neither extract inhibited the growth of the fungus (Figure 4C). This indicates that anthocyanins do not suppress the growth of *B. cinerea* directly and that the resistance requires living host cells. Between 24 and 48 hr after infection with *B. cinerea*, lesions on red fruit spread quickly, while on purple fruit their size remained small (Figure 1E). 3,3′-diaminobenzidine (DAB) staining of H_2_O_2_ in infected red and purple fruits during this period showed that a ROS burst was generated at the infection site. However, the ROS burst on red fruit spread widely, whereas on purple fruit strong ROS induction was restricted to the inoculation site (Figure 4D). The oxidative burst is thought to potentiate infection by necrotrophic pathogens that feed on dead tissue, facilitating the expansion of disease lesions [26–28]. Vacuum infiltration of diphenyleneiodonium chloride (DPI), an NADPH oxidase inhibitor, into red fruit prior to *B. cinerea* inoculation restricted the spread of lesions, whereas infiltration of purple tomatoes with glucose and glucose oxidase (which induce ROS, through the generation of H_2_O_2_) increased lesion growth in purple fruit (Figure 4E). These data suggest that in purple tomatoes, anthocyanins alter the dynamics of the ROS burst generated by *B. cinerea* infection and limit the induction of cell death necessary for growth of the necrotroph.

In addition to their high nutritional value [14], anthocyanin-rich purple tomatoes have 2-fold longer shelf life, the combined result of increased resistance to opportunistic pathogens and slower ripening at late stages. These traits are associated with the accumulation of anthocyanins in tomatoes. Anthocyanins specifically alter the spread of the ROS burst generated as part of necrotrophic infection and so reduce susceptibility to *B. cinerea.* Accumulation of anthocyanins results in high hydrophilic antioxidant capacity, which reduces the increase in ROS levels, that occurs late in fruit development, and the reduction in ROS may suppress the later stages of ripening (Figure 4F). The association of slower ripening with elevated hydrophilic antioxidant capacity of fruit offers new, yet broad, targets for breeders to extend the postharvest shelf life of fruit. Additionally, anthocyanins could be used to reduce the susceptibility of ripe fruit specifically to *Botrytis cinerea*, the most important fungal pathogen of soft fruit.

## Accession Numbers

The GEO accession number for the TOM2 microarray data reported in this paper is GSE46341.

## Supplemental Information

Document S1. Supplemental Experimental Procedures, Figures S1–S4, and Table S1

Data Set S1. List of Genes that Showed >3-Fold Differences in Transcript Levels between Purple and Red Sectors of VIGS-Del/Ros1-Silenced Fruit over at Least Two Time Points, Related to Figure 2
