# Supplementary material for: Anthocyanins Double the Shelf Life of Tomatoes by Delaying Overripening and Reducing Susceptibility to Gray Mold
Source: Curr Biol. 2013 Jun 17;23(12):1094–100. doi: 10.1016/j.cub.2013.04.072 (PMC3688073; doi:10.1016/j.cub.2013.04.072)
Supplement: Document S1. Supplemental Experimental Procedures, Figures S1–S4, and Table S1 [file mmc1.pdf]

Current Biology, Volume *23*

Supplemental Information

Anthocyanins Double the Shelf Life of Tomatoes by Delaying Overripening and Reducing Susceptibility to Gray Mold

Yang Zhang, Eugenio Butelli, Rosalba De Stefano, Henk-jan Schoonbeek, Andreas Magusin, Chiara Pagliarani, Nikolaus Wellner, Lionel Hill, Diego Orzaez, Antonio Granell, Jonathan D.G. Jones, and Cathie Martin

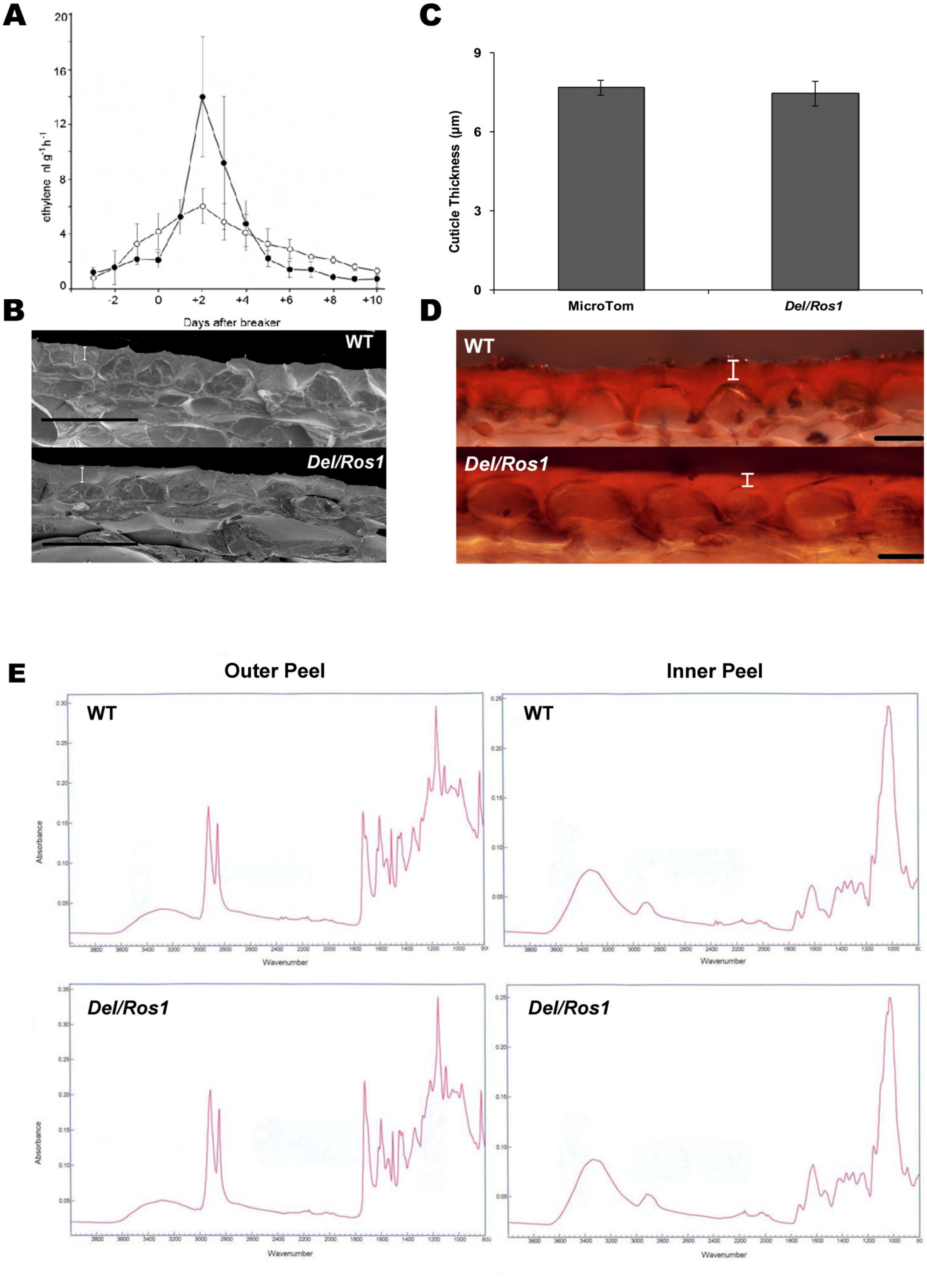
A
ethylene nl g-1 h-1
Days after breaker
B
WT
Del/Ros1
C
Cuticle Thickness (µm)
MicroTom
Del/Ros1
D
WT
Del/Ros1
E
Outer Peel
Inner Peel
WT
Del/Ros1
Absorbance
Wavenumber

Figure S1. Extended shelf life in purple tomato is not due to impaired ethylene production nor changes of cuticle thickness or composition, Related to Figure 1

(**A)** Ethylene production in red (○) and purple (●) tomato fruit during ripening. Data represent mean values ± se of at least ten individual fruits for each genotype.

(B) Freeze-fracture scanning electron microscopy (SEM) indicated no significant morphological changes to the peel of purple fruit. Arrows indicate cuticle thickness. Scale bars represent 50µm.

(**C**) Cuticle thickness of purple and red tomatoes. Measurements were made above the centre of each epidermal cell as indicated by bars in (**D**). Error bars show the standard error of the mean ($n \geq 3$).

(**D**) Light micrographs of sections of the fruit surface of tomato stained with Sudan Red. Arrows indicate cuticle thickness. Scale bars show 20 µm.

(**E**) FT-IR (Fourier Transformed Infra Red) spectra of wild type (**top**) and *Del/Ros1* (**bottom**) tomato peel. The analysis of the outer (**left**) and inner side (**right**) of the peel is shown.

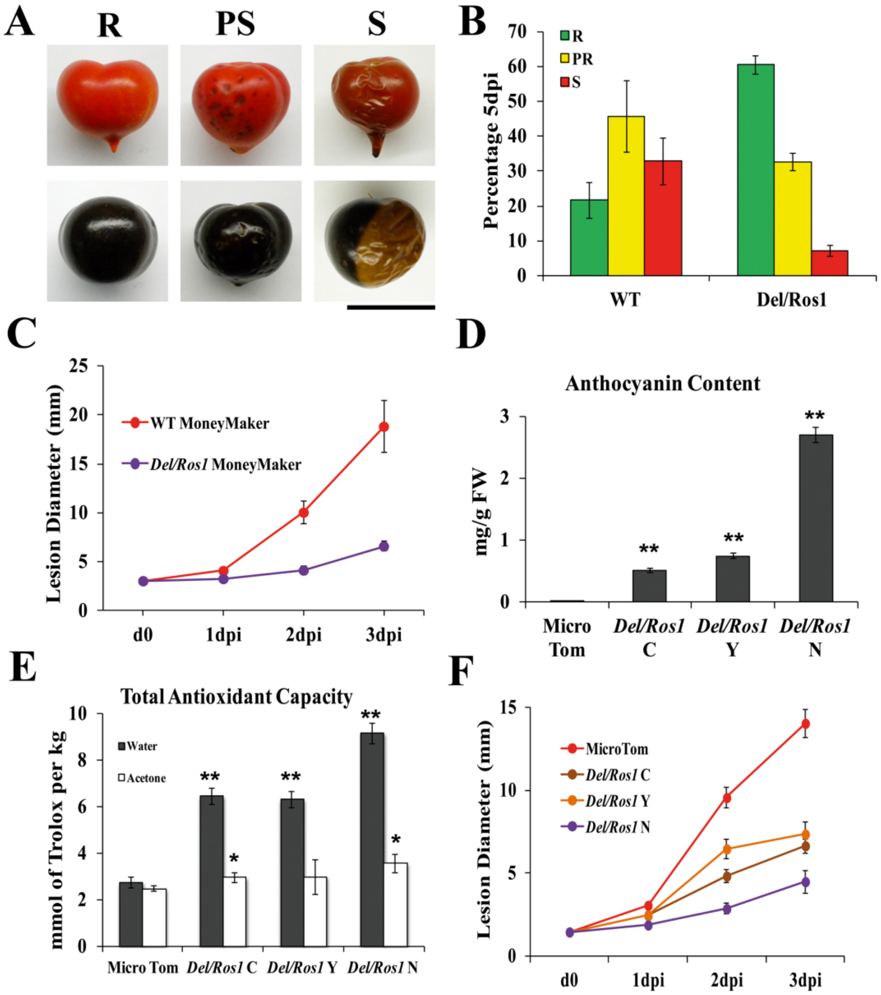

Figure S2. Enrichment of anthocyanins in tomato fruit reduces susceptibility to *Botrytis cinerea*, Related to Figure 1

(**A**) Phenotypes of fruits showing resistance (**R**), partial susceptibility (**PS**) and susceptibility (**S**) when sprayed with spores of *B.cinerea*, pictures were taken at 5dpi. All scale bars represent 2 cm.

(**B**) Different degrees of susceptibility to *B. cinerea* shown by red and purple fruit in spraying tests. Fruit were checked 5 days after inoculation. Error bars represent the standard error of the mean for three independent assays. Resistance (**R**), partial susceptibility (**PS**) and susceptibility (**S**)

(**C**) Lesion development following *Botrytis* inoculation of red and purple Moneymaker (normal size variety) fruits. Error bars indicate the standard error of the mean ($n \geq 3$).

(**D**) Anthocyanin contents in different transgenic MicroTom lines. Error bars represent the standard error of the mean ($n=3$). ** ($p<0.01$) compared with WT, red fruit.

(**E**) Trolox equivalent total antioxidant capacity of different transgenic lines. Error bars represent the standard error of the mean ($n=3$). * ($p<0.05$) and ** ($p<0.01$) compared with WT, red fruit.

(**F**) Lesion development in different transgenic fruit lines following *B.cinerea* inoculation. Error bars show the standard error of the mean ($n=3$)

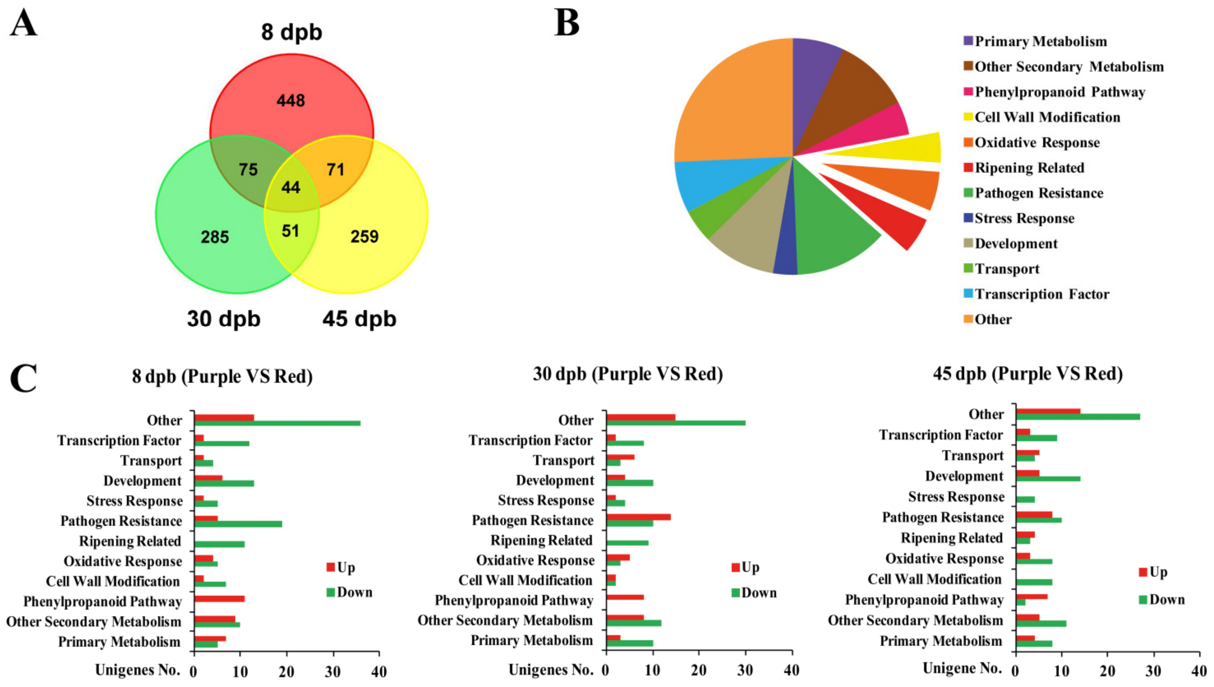

Figure S3. Ripening-related genes were suppressed in anthocyanin-enriched tomatoes, Related to Figure 2

(**A**) 241 genes were selected showing>3-fold differences in transcript levels between the purple and red sectors of VIGS-*Del/Ros1* silenced fruit over at least two time points.

(**B**) Functional annotation of the 241 genes showing >3-fold differences in expression between purple and red sectors over at least two time points.

(**C**) Functional classification of selected differentially-expressed genes at 8 days post breaker (dpb), 30dpb and 45 dpb.

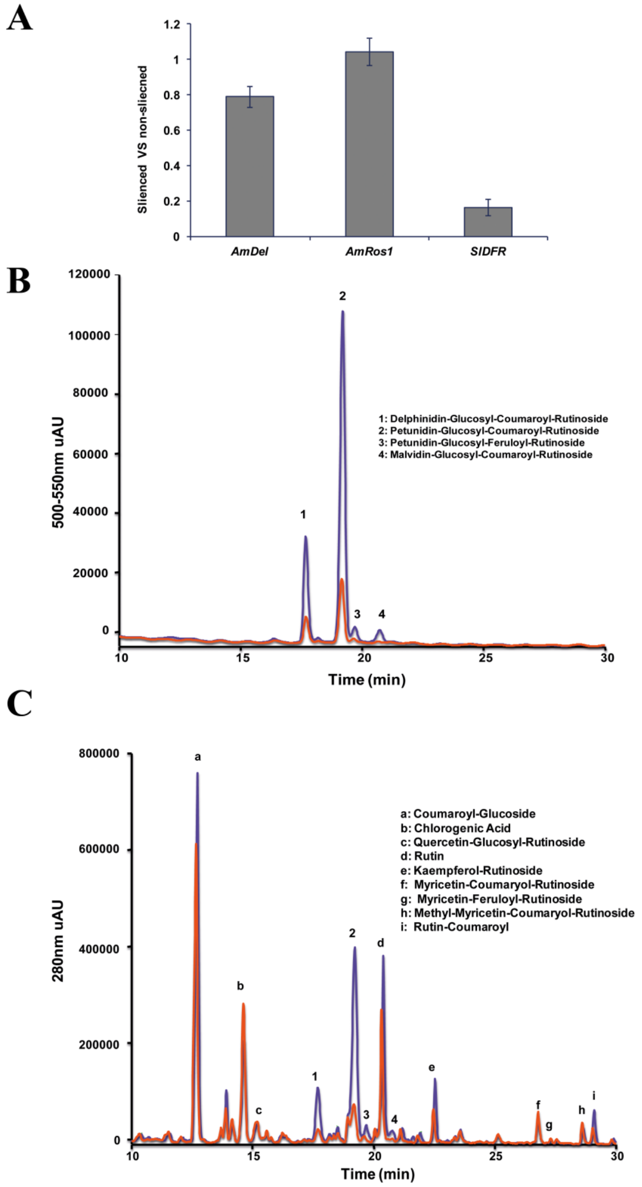

Figure S4. Silencing of *SlDFR* in purple tomato by VIGS substantially reduces anthocyanin accumulation but maintains elevated levels of other phenolic compounds, Related to Figure 3

(**A**) Comparison of gene expression (determined by qRT-PCR) in silenced and non-silenced sectors of VIGS-*SlDFR* fruit. Red and purple sectors showed similar expression of *Del* and *Ros1*, whereas *SlDFR* expression was significantly reduced in silenced sectors. Error bars show standard error of the mean, n=3.

(**B**) Comparative HPLC analysis of methanol extracts of tomato fruit recorded at 500-550 nm showing that accumulation of anthocyanin compounds was reduced in VIGS-*SlDFR*-silenced sectors (orange line) compared to non-silenced sectors (purple line).

(**C**) Comparative HPLC analysis of methanol extracts of tomato fruit at 280 nm indicates, aside from more than 80% reduction in anthocyanin levels (peaks 1-4, the same as in (**B**)), there were no other significant differences in levels of other phenolic compounds between VIGS-*SlDFR*-silenced (orange line) and non-silenced (purple line) sectors.

Table S1. Primers used in RT-qPCR, Related to Figure 2

Gene	Locus	Primer sequence (5'-3')
SlUBI	Solyc01g056940	F: GCCAAAGAAGATCAAGCACA<br>R: TCAGCATTAGGGCACTCCTT
SlPG2a	Solyc10g080210	F: ATCTGGACAAGCTAGCAACATCAA<br>R: TATACATGGTTCAACTCGATCACAA
SlTBG4	Solyc12g008840	F: CTTGGCGAAACAGAAATGGT<br>R: ACCTCGAACCCATTCAACAG
SlPSY	Solyc03g031860	F: TGTTGGAGAAGATGCCAGAA<br>R: TTTATCGGTCACCCTTCCAG
AmDel	M84913	F: AGAAAACACGGGTGTCCAAG<br>R: CGTCGACTTTCCTCTCAAGC
AmRos1	DQ275529	F: AAAAGAATTGTCGTGGAGTGAGA<br>R: ATCATTGTAAAATTGCGTTTGCT
SlDFR	Solyc02g085020	F: GACTTGCCGACAGAAGCAAT<br>R: GTGCATTCTCCTTGCCACTT

Supplemental Experimental Procedures

Storage tests

WT (red) and *Del/Ros1* (purple) MicroTom fruits were harvested at 14 days post breaker (d0=14dpb). All fruits were sterilized in 10% bleach for 10 minutes, followed by rinsing three times in sterilized water and air-drying. Ten fruits were placed in one sterilized glass jar and kept at 18°C in the dark. Every week, the total weight of 10 fruits was measured and fruits showed softening and collapse symptoms were assessed. After each measurement, fruits were transferred to a new sterilized jar.

Texture analysis

Mechanical tests were carried out with a Stable Microsystems TaXT2 texture analyzer. A 0.5 mm diameter probe with a 45° conical tip was attached to the crosshead of the test machine. Skin penetration tests were carried out with a test speed of 0.1 mm/sec and a maximum penetration depth of 3 mm. Fruits were held in a small cup between two metal plates on the sample table.

The force-distance plots show typically two distinct regions. At the beginning there was an approximately linear increase of the force up to the bioyield point. At this point the skin was penetrated and the force reading suddenly dropped from its local maximum (fmax). After that, a second gradient was observed, which represented the penetration of the probe into the flesh.

The bioyield force (MPa) = $f_{max}/(\pi R^2)$

R equals the radius of probe (0.25 mm)

To obtain the firmness of the fruit skin, the slope= $(f_{max}-f_0)/d_{max}$, (with: initial force (f_0), the bioyield force at the local maximum (fmax) and at distance (d_{max})) was divided by the area of the flat end of the probe tip.

The firmness of fruits (MPa/mm) was calculated as $(f_{max}-f_0)/(d_{max}\times \pi R^2)$

Scanning electron microscopy of tomato cuticle

Blocks of fruit pericarp tissue including peel were frozen in nitrogen slush at –190°C. Frozen samples were warmed to -100°C prior to fracture, and the specimens were then sputter-coated with platinum and examined in a Philips XL 30 FEG scanning electron microscope (SEM) fitted with a cold stage.

Measurements of cuticle thickness

Cuticle thickness measurements [1] involved slicing WT and purple fruit into 10-30 µm thick sections. Sections were stained with Sudan red and thickness was determined using a Leica DM6000 microscope, taking the average of 8-10 measurements (**Figure 2D**). The average and standard error of the mean of three to five biological replicates were recorded.

FT-IR (Fourier Transform Infra-Red) spectroscopy of tomato peel

Tomato peel was obtained from ripe wild type and *Del/Ros1* tomato fruit carefully removing any attached flesh material. The material was washed sequentially with 1% (w/v) SDS in 50 mMTris-HCL pH 7.2, water, 50% ethanol, acetone and then air dried at room temperature. FT-IR spectra were recorded on a BioRad FTS175C (BioRad, now Varian) spectrometer equipped with a MCT detector and a Golden Gate single-reflection diamond ATR sampling accessory (Specac). Both the outer and inner sides of the peel were measured. The dry samples were gently pressed onto the ATR crystal, with either the inside or outside in contact with the crystal. For each spectrum, 128 scans at 2 cm^{-1} resolution were averaged and referenced against the empty crystal.

Botrytis cinerea infection

B.cinerea (B05.10) was collected as described previously [2]. Red and purple tomatoes were harvested 14 days after breaker and surface sterilized. Intact red and purple fruits were sprayed thoroughly with spores (2.5×10^5 spores/mL) three times in the fume cabinet and kept at 20°C, in high humidity. Infection symptoms were observed at 3dpi, 4dpi and 5dpi.

For wound inoculation, the fungal culture was diluted with medium to 5×10^4 spores/mL (for MicroTom fruit) or 1×10^5 spores/mL (for MoneyMaker fruit) and inoculated for 1.5 h to stimulate germination. The spore innoculum (5µL) was added to each wound. Lesion diameter was measured 24, 48 and 72 hours after inoculation. To quantify *Botrytis* growth using qPCR, half infected MicroTom fruits were harvested three days after inoculation. Seeds were removed from fruit samples which were then freeze dried. Tissues were homogenised with aTissueLyser and total DNA was extracted using Qiagen DNeasy Plant Mini Kit. DNA (50ng) was used for qPCR and the ratio of *B.cinerea Cutinase A*gDNA to tomato *ACTIN* gDNA was measured. The primers designed for genomic DNA were *SlACT*-q-F (ACAACTTTCCAACAAGGGAAGAT), *SlACT*-q-F (TGTATGTTGCTATTCAG GCTGTG), *BcCutA*-q-F (ATTCCACAATATGGCATGAAATC) and *BcCutA*-q-R (ATGTTATCTC ATGTTATCTC).

Growth tests of *B.cinerea* were performed on PDA plates. PDA medium was made up with 50% red or purple tomato juice, prepared by homogenising whole fruit and then centrifugation. As a negative control, 15mg/mL Triademinol was added to PDA medium. Blocks of B05.10 mycelium (5mm diameter) were cut from a *B.cinerea* plate and placed in the cente of the test plates. Mycelial growth was measured daily. Streptomycin and ampicillin (100mg/L) were added to all the plates to prevent infection by other fungi or bacteria, respectively.

Virus Induced Gene Silencing (VIGS)

TRV-based silencing vectors pTRV1, pTRV2 and pTRV2-*Del/Ros1* were prepared as recorded previously [3].271bp of *SlDFR* cDNA was inserted into a pTRV2 gateway vector. Primers for gateway cloning were SlDFR-attB1(GGGGACAAGTTTGTACAAAAAAGCAGGCTTAATTGATTTCATTAGCATCA) and SlDFR-attB2 (GGGGACCACTTTGTACAAGAAAGCTGGGTACTGGCCATTTCTGTCGCAC). Agroinfiltration of *Del/Ros1* MoneyMaker fruit was performed by syringe [3].

Microarrays

VIGS-*Del/Ros1* silencing in *Del/Ros1* MoneyMaker fruit was undertaken at mature green stage. Fruit were tagged at breaker. Total RNA was extracted from the red and purple sectors on individual VIGS-*Del/Ros1* fruits at 8, 30 and 45 days after breaker. The changes in transcript profiles were compared between the red and purple sectors on the same fruit. For each time point, three biological replicates were used. All microarray data are available at the Gene Expression Omnibus under accession number GSA46341.

The TOM2 array was used to monitor changes in transcript levels. [4]. RNA amplification and aminoallyl labeling were performed by using the Message Amp™ aRNA kit (Ambion # AM1750). Microarray hybridisation, scanning and data analyses were performed as described previously[5]: Telechem Hybridization Chambers (Corning) were employed for the manual hybridisation of labelled samples to the TOM2 long-oligo, 11,862 - gene, microarray. A GenePix 4000B scanner was used for scanning the microarray slides at 532 nm and 635 nm; with a resolution of 10 µm and 100% power. Images were quantified using GenePix Pro 4.1 image analysis software (Axon Instruments/Molecular Devices). Valid spots were defined as having intensity values ≥2-fold the mean background intensity in ≥1 channel.

Normalisation and calculation of differential expression were performed in R (http://cran.r-project.org) using Bioconductor libraries (http://www.bioconductor.org). Within-array normalisation was performed with the aim of making the background-subtracted log-ratios average to zero within each microarray. This was achieved by fitting a LOESS curve to each print tip (with the parameters smoothing filter = 0.4, iterations = 3; and $\delta = 0.01$). The resulting data were then subject to a between-array normalisation step where the average intensity values for each array were transformed such that they followed the same empirical distribution; while leaving the log-ratios unaffected. Differential expression was calculated by fitting a linear model for each gene across the microarrays[6] (where the contrasts were parameterised as differential gene expression between purple and red sectors, at 8, 30 and 45 days post-breaker respectively); the estimated coefficients were, in turn, computed from the fit; followed by computation of moderated *t*-scores and log-odds by empirical Bayes shrinkage of the standard errors towards a common constant. Genes exhibiting a fold-change of ≥3 and Benjamini Hochberg-adjusted *p*-values ≤ 0.05 were selected for further scrutiny.

RT-qPCR

RNA extraction and cDNA synthesis were conducted as described previously [7]. Primers for RT-qPCR are listed in **Table S1**.

Cell wall degrading enzyme activities

Red and purple tomatoes were washed, deprived of seeds and homogenized in liquid nitrogen. Homogenized sample (1g) was extracted with 3 mL of sodium acetate buffer (50 mM, pH 5.5, 1 M NaCl, 10 g/L polyvinyl-polypyrrolidone; PVPP) under agitation for 2h at 4°C, centrifuged at 1000 x g for 10 min and the supernatant collected. The supernatant then was dialyzed against sodium acetate buffer (50 mM, pH 5.0) overnight at 4°C[8]. PG activity was measured in a mixture containing 50 mM sodium acetate buffer pH 5.0, 0.15% (w/v) polygalacturonic acid, and 1 mL of enzyme extract, in a total volume of 3mL. The mixture was incubated at 37°C, aliquots of 300 µL were taken at different times, mixed with 1mL Borate buffer pH9 and 200µL 1% 2-cyanoacetamide (Sigma-Aldrich). To stop the reaction, the mixture was immersed in a boiling bath for 10 mins. Samples were cooled to room temperature and the OD 276 nm was measured. Results were expressed as delta OD in 1s under the assay conditions per kilogram of fresh fruit. β-Galactosidase (TBG) reaction mixture consisted of 0.5 mL of 0.1 M citrate (pH 4.0), 0.4 mL of 0.1% BSA, 0.1 mL of enzyme extract, and 0.5 ml of 10 mM p-nitrophenyl- β-galactoside. After 15 min at 37°C, the reactions were terminated by the addition of 2 mL of 0.4 M sodium carbonate, and the liberated p-nitrophenol was measured at 420 nm[8]. Results were expressed as delta OD in 1s under the assay conditions per kilogram of fresh fruit.

Malondialdehyde measurements

Fruit pericarp (2.5g) was homogenised in 10 mL of 10 mM sodium phosphate buffer, pH 7.2 and then centrifuged at 2000g for 10 min. A sample of supernatant (100µL) was added to a 2mL tube containing 0.4mL of distilled water, 0.25 mL of 20% (w/w) trichloroacetic acid, and 0.5 mL of 10 mM thiobarbituric acid. A control was run for each sample in which thiobarbituric acid was replaced by an equal volume of distilled water. The mixture was heated in a boiling water bath for 30 min and then centrifuged for 10 min at 2000g to remove haziness. The cleared samples were allowed to equilibrate to room temperature before the absorption at 532 nm was measured. The concentration of malondialdehyde (MDA) was calculated using its molar extinction coefficient of 156 mM^{-1} cm^{-1}.

Analysis and identification of anthocyanins and other phenylpropanoids

Freeze-dried tomato tissue (1g) was extracted with 50mL 80% MeOH overnight. Extracts were cleared by filtration through paper and then through a 0.22 mm membrane filter (Millipore). Aliquots (30µL) of the original sample were then diluted with 270µL 20% MeOH and centrifuged. Finally 20µL of the diluted sample were added to 180µL 20% MeOH in flat-bottomed inserts.

All samples were analysed on a Surveyor HPLC system attached to a DecaXPplus ion trap MS (both Thermo), using 10µL injections. Phenolics were separated on a 100×2mm 3µ Luna C18(2) column (Phenomenex) using the following gradient of acetonitrile versus 0.1% formic acid in water, run at 300$\mu L.min^{-1}$ and 30°C: 0 min, 1% ACN; 4 min, 1% ACN; 23 min, 30% ACN; 30 min, 70% ACN; 30.5 min, 1% ACN; 37 min, 1% ACN.

Phenolics were detected by light absorbance, collecting full spectra from 200-600 nm, and chromatograms at 280 nm and 500-550 nm both with 19nm band width. For positive electrospray detection, spectra from *m/z* 100-2000 and data-dependent MS2 of the most abundant precursor ions at collision energy of 35% and an isolation width of *m/z* 4.0 were collected. Dynamic exclusion was used to ensure that after two spectra had been collected for a precursor ion, it would be ignored for 0.5min in favour of the next most abundant ion. Spray chamber conditions were 50 units sheath gas, 5 units aux gas, 350°C capillary temperature, and 3.8kV spray voltage using a steel needle kit.

Statistics

All experiments were repeated and yielded reproducible results. The most representative data are shown in this paper. Data are presented as means ± standard error of the mean, unless stated otherwise. To compare group differences, paired or unpaired, two-tailed Student's *t*-tests were used. *p* values less than 0.05 were recognized as significant.

Supplemental References

1. Yeats, T.H., Buda, G.J., Wang, Z., Chehanovsky, N., Moyle, L.C., Jetter, R., Schaffer, A.A., and Rose, J.K. (2012). The fruit cuticles of wild tomato species exhibit architectural and chemical diversity, providing a new model for studying the evolution of cuticle function. Plant J *69*, 655-666.

2. Stefanato, F.L., Abou-Mansour, E., Buchala, A., Kretschmer, M., Mosbach, A., Hahn, M., Bochet, C.G., Metraux, J.P., and Schoonbeek, H.J. (2009). The ABC transporter BcatrB from Botrytis cinerea exports camalexin and is a virulence factor on Arabidopsis thaliana. Plant J *58*, 499-510.

3. Orzaez, D., Medina, A., Torre, S., Fernandez-Moreno, J.P., Rambla, J.L., Fernandez-Del-Carmen, A., Butelli, E., Martin, C., and Granell, A. (2009). A visual reporter system for virus-induced gene silencing in tomato fruit based on anthocyanin accumulation. Plant Physiol *150*, 1122-1134.

4. Bugos, R.C., Chiang, V.L., Zhang, X.H., Campbell, E.R., Podila, G.K., and Campbell, W.H. (1995). RNA isolation from plant tissues recalcitrant to extraction in guanidine. Biotechniques *19*, 734-737.

5. Lytovchenko, A., Eickmeier, I., Pons, C., Osorio, S., Szecowka, M., Lehmberg, K., Arrivault, S., Tohge, T., Pineda, B., Anton, M.T., et al. (2011). Tomato fruit photosynthesis is seemingly unimportant in primary metabolism and ripening but plays a considerable role in seed development. Plant Physiol *157*, 1650-1663.

6. Smyth, G.K. (2004). Linear models and empirical bayes methods for assessing differential expression in microarray experiments. Statistical applications in genetics and molecular biology *3*, Article3.

7. Luo, J., Butelli, E., Hill, L., Parr, A., Niggeweg, R., Bailey, P., Weisshaar, B., and Martin, C. (2008). AtMYB12 regulates caffeoyl quinic acid and flavonol synthesis in tomato: expression in fruit results in very high levels of both types of polyphenol. Plant J *56*, 316-326.

8. Rodoni, L., Casadei, N., Concellon, A., Chaves Alicia, A.R., and Vicente, A.R. (2010). Effect of short-term ozone treatments on tomato (Solanum lycopersicum L.) fruit quality and cell wall degradation. J Agric Food Chem *58*, 594-599.
